# Supplementary material for: Causal Relationship between Aging and Anorexia Nervosa: A White-Matter-Microstructure-Mediated Mendelian Randomization Analysis
Source: Biomedicines. 2024 Aug 16;12(8):1874. doi: 10.3390/biomedicines12081874 (PMC11351342; doi:10.3390/biomedicines12081874)
Supplement: Supplementary file 1 [file biomedicines-12-01874-s001.zip › Supplementary Figure/Figure S9/Leaveoneout plot of MR analysis for ubm-b-1836 to AN.pdf]

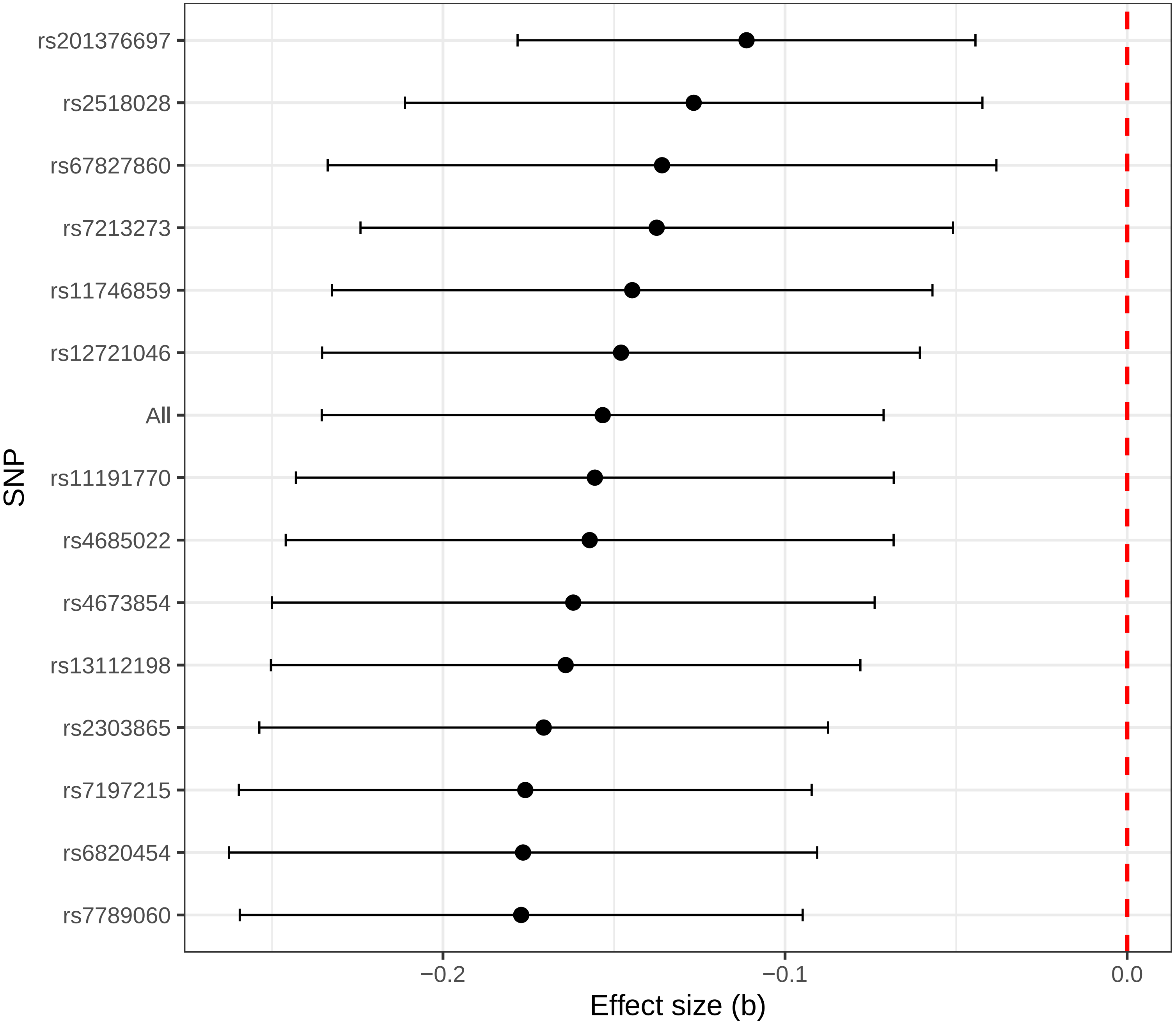
rs201376697
rs2518028
rs67827860
rs7213273
rs11746859
rs12721046
All
rs11191770
rs4685022
rs4673854
rs13112198
rs2303865
rs7197215
rs6820454
rs7789060
SNP
−0.2
−0.1
0.0
Effect size (b)
